# County-level mobility and sociopolitical context in the spread of COVID-19 during spring 2020

**DOI:** 10.1007/s10729-025-09722-w

**Published:** 2025-10-04

**Authors:** Chris Parker, Jorge Mejia

**Affiliations:** 1 Darden Graduate School of Business, University of Virginia, 100 Darden Boulevard, Charlottesville, VA 22903 USA; 2 Department of Operations & Decision Technologies, Kelley School of Business, HH4100, 1309 E. 10th St., Bloomington, IN 47405 USA

**Keywords:** COVID-19, Open data, Sociopolitical, Managing pandemics, Econometrics, Longitudinal analysis, Panel data

## Abstract

The implementation of social distancing policies is key to reduce the spread of the recent COVID-19 pandemic and future pandemics. However, their effectiveness ultimately depends on human behavior. For example, in the United States, compliance with social distancing policies widely varied in Spring 2020. What factors were associated with the observed variability in behavioral compliance with the policies? Utilizing detailed county-level data, we estimate the association between human mobility and the growth rate of COVID-19 cases across approximately 3,100 U.S. counties from January 1, 2020 to June 20, 2020. In addition, using data from U.S. presidential elections we measured how the association between mobility and COVID-19 growth rate varied as a function of county voting pattern. Our results generalize previous reports in finding a significant association between political leaning and the COVID-19 growth rate. These results highlight how it might be beneficial to consider political orientation when building models of the multivariate relationships between the spread of pandemics and public health policies intended to curb the expansion of the pandemic.

## Highlights

- Political views can influence the ability for policy makers to respond to a pandemic.
- Political views might have played a role in influencing human response to the U.S. national travel ban order and, in turn, impacted the spread of the pandemic.
- Future pandemic response should consider the local political environment when deciding when and how to implementing policy.

## Introduction

During the early spreading of the COVID-19 pandemic, US federal and local governments put in place policies that suggested changes to human behavior deemed critical to curtailing the spread of the virus. Such interventions included using masks and the promotion of physical distancing between individuals [13, 19]. However, adherence to government policies varied significantly across the United States, especially during the first wave of the pandemic in the spring of 2020. Major differences in the public rhetoric used to describe the pandemic, and individuals’ responses to the pandemic, may have been at least partially associated with differences in predicted risks due to COVID-19. For example, it was found that in the early stage of the pandemic, partisanship was reported to be the single most important predictor of face mask use [41].

Here, we test whether county-level political leaning was associated with social-distancing behavior and the growth rate of COVID-19 cases. It is worth noting that we refrain from discussing or making causal claims between any of the variables used in the analysis, given the secondary data used in the study and the lack of any plausibly exogenous variation.

Early in 2020, U.S. local, state, and federal agencies imposed orders mandating varying degrees of social distancing [31]. For example, these policies included restrictions to workplace practices, public gatherings, and travel. Recent research has shown that differences in the speed and magnitude at which policies were imposed or relaxed by governments correlated with the spread of COVID-19 and impacted the local economies [27, 32, 60]. In some states, the implementation of these orders and policies was complemented by efforts to communicate the severity of the pandemic and persuade citizens to adapt their behavior. For instance, New York State officials used televised briefings, watched by millions, to inform citizens about the dangers of COVID-19 [35]. On the contrary, other state officials, such as ones from Florida, consistently downplayed the severity of the pandemic [10] and associated health risks. An in-depth evaluation of the epidemic process requires a deeper understanding of how government policies and efforts to engage with citizens, may ultimately influence human behavior and response [20, 59]. Indeed these strategies (i.e., mandates, and direct communications from officials) are deeply interrelated because the success of the policies in reducing the spread of COVID-19 is likely to depend on the degree to which individuals comply with the mandates and social norms. After all, in the view of many U.S. public officials, it would not be possible to enforce compliance with all social distancing orders.

We investigated whether the political leaning of individual counties within the U.S. was associated with the growth rate of COVID-19 cases during the Spring of 2020. To do so we combined several open data sources: the University of Maryland’s COVID-19 Impact Analysis Platform [40] (referred to as the UMDC19 dataset), Massachusetts Institute of Technology’s Election Data and Science Lab [43] (referred to as the MITED dataset), numerous American Community Survey tables (jointly referred to as the ACS dataset), and U.S. Department of Agriculture data measuring urban/rural counties. Together these datasets allowed us to investigate how individuals’ mobility behavior changed early in the pandemic and as a function of the distancing orders implemented by the U.S. governments. The UMDC19 data contained a number of county-level metrics. We utilize the cumulative total number of COVID-19 cases to calculate the COVID-19 case growth rate and the social distancing index to construct a measure of individual mobility. The data is at the county-day level, and we utilize data from January 1, 2020, to June 20, 2020. The MITED data contains county-level voting results from the 2016 presidential election. Combining these two datasets allows us to investigate three relationships. First, increased levels of individual mobility are associated with high levels of COVID-19 case growth rates. Second, in early 2020, there was generally no relationship between political leaning and individual mobility, but a strong positive relationship developed in around the March 14, 2020 announcement of travel restrictions to the U.S., which we refer to as the national travel ban. Finally, there is an association between political leaning and the differentiation in the COVID-19 growth rates before and after the national travel ban. We combine the ACS and Department of Agriculture data to demonstrate robustness of these effects. Our results corroborate previous reports and extend the understanding that there are relationships between political narratives, human behavior, and public health outcomes during the ongoing pandemic [4, 7, 15, 21, 26, 61].

Previous reports have used spreading models [59] to predict the impact of government policies to limit mobility on the extent and effects of the COVID-19 pandemic [1, 36]. For example, using spreading models, it has been shown that the effective implementation of social distancing policies is associated with reductions in the growth rate of COVID-19 cases, with an average reduction ranging from 3.0% during the early onset days of social distancing measures to 8.6% in the later days [18]. Although our results cannot express causal relationships, they can inform researchers about currently underutilized variables that may improve the accuracy of models of spreading during a pandemic [59]. Our hope is that the inclusion of these variables can reduce the effects of inaccurate data on the evaluation of mitigation policies [46].

Furthermore, our results validate and extend previous research that has utilized non-traditional variables to inform COVID-19 response including detailed mobile phone usage data which has been widely used throughout the world including in specific demonstrations of value in Ghana [37], Gambia [6], France [17], Sweden [2], and The Congo [28], as well as broader multi-country studies in Europe [39, 58] and a broad swath of developing countries [42]. Additional examples include Twitter data [45], and personal loan data [38]. More examples and summaries are available in [11]. It also highlights the importance of data experts working with governments to identify data needs and evaluate the impact of interventions. This digital governmental transformation will be key to fighting future pandemics [22]. In addition, our results can be used as part of the discussion around the need for data privacy and the value of data in fighting pandemics [3, 12, 33, 58].

## Data sources

Numerous datasets were used for this analysis. Below we describe each dataset and how it contributed to the final county-day dataset. All data is available on Open Science Framework (OSF); see the data availability statement for a link and details.

### University of Maryland COVID-19 Data (UMDC19)

This data was downloaded from https://data.covid.umd.edu/ [40] on June 24, 2020, and contains data from January 1, 2020, until June 20, 2020. We primarily use the county-level data restricted to only the continental United States (Alaska and Hawaii were excluded from all of the analyses because we encountered problems matching counties to the other datasets). More specifically, the columns used from the UMDC19 dataset were: the county name, county- and state-level Federal Information Processing System (FIPS) codes, number of new COVID-19 cases, population, social distancing index, population density, and testing capacity. The creation and measurement of the social distancing index is a crucial component of the University of Maryland COVID-19 Impact Analysis Platform which describes the calculation of the metric as

“The social distancing index is computed from six mobility metrics by this equation: social distancing index = 0.8*[% staying home + 0.01*(100 - %staying home)*(0.1*% reduction of all trips compared to pre-COVID-19 benchmark + 0.2*% reduction of work trips + 0.4*% reduction of non-work trips + 0.3*% reduction of travel distance)] + 0.2*% reduction of out-of-county trips. The weights are chosen based on share of residents and visitor trips (e.g., about 20% of all trips are out-of-county trips, which led to the selection of a weight of 0.8 for resident trips and 0.2 for out-of-county trips); what trips are considered more essential (e.g., work trips more essential than non-work trips); and the principle that higher social distancing index scores should correspond to fewer chances for close-distance human interactions and virus transmissions.”

Interested readers can read more details about the social distancing metric at the COVID-19 Impact Analysis Platform website: https://data.covid.umd.edu/methods/index.html.

To enable interpretation of the social distancing index, we create the individual mobility metric as 100 minus the social distancing index and the lag individual mobility metric as the individual mobility metric in a county but from two weeks (fourteen days) prior. For consistency, we also lag the testing capacity to reflect the testing capacity at the same time as the mobility. The two week assumption aligns with the incubation period. Nevertheless, we run applicable analyses with a seven-day lag and a 21-day lag finding similar results. In order to create the growth rate variable, we first calculate the cumulative number of cases up to a given day. The case growth rate is then ln(*cumulative*_*cases*_*today*+1) –ln(*cumulative*_*cases*_*yesterday*+1) . This use of the change in the natural log results in a percentage change which allows for direct comparison across counties of different populations. One was added to the cumulative number of COVID-19 cases to account for issues when the cumulative number of cases is zero, which would make the log undefined. We adjust the population to be measured as a population in thousands by dividing the population by 1000. We only use the state-level data to bring in the state name, which is not included in the county-level data. We merge the state- and county-level datasets based on the state FIPS code, which is present in both files.

### Massachusetts Institute of Technology Election Data (MITED)

In order to demonstrate the robustness of our findings, we use two different approaches to election data. First, we use data that would have been available at the time of the pandemic which means election data from the 2016 presidential election. As an alternative, we use the 2020 presidential election data. This data would not have been available in the spring of 2020 since the election was not until November, but it may be a better measure of the political leanings of counties than the 2016 data.

Both 2016 and 2020 election data were downloaded on February 13, 2025 from [43]. We use the candidate, candidate votes, total votes, year, and FIPS columns in this dataset. We exclude counties that do not have a FIPS code since they cannot not be merged with the UMD dataset. Examples include the Uniformed and Overseas Citizens Absentee Voting Act (UOCAVA) in Maine, Rhode Island Federal Precinct, and Connecticut Statewide write-in. The percent of votes for each candidate is calculated as 100 times the number of votes for that candidate divided by the total votes for Republican and Democratic candidates. The Republican 2016 (2020) Margin is then the percent of votes for the Republican candidate in a county minus the percent of votes for the Democratic candidate in a county. The data is then merged with the UMD data based on the county FIPS code. Oglala Lakota County in South Dakota does not properly merge and is dropped from the analysis.

### ACS Data (ACS)

In Section 3.3.1, we include county-level controls to ensure the correlations we observe are robust. As with the election data, we use data from two different time periods for this purpose. First, we use ACS data available in March 2020 which is the 2018 version of data with estimates derived from 2014-2018. This data was released in December 2019 and was downloaded on April 7, 2025. Second, we use the 2020 ACS data with estimates derived from 2016-2020 which was not released until December 2021, but should have a more accurate representation of the country at the time of the pandemic. The 2020 version of ACS data was downloaded on March 4, 2025. Below we describe the specific tables used. For each table we discuss the variables used. Tables were merged using the GEO_ID column which was then deconstructed into the county-level FIPS for connecting to other datasets. Note that we used the ACS data instead of decennial census data as the move to the short form for the decennial census means it no longer collects the control variables we were seeking. for such as education and employment. See [55] for additional information.

#### Education and Employment Data (EdEmD)

Data on education and unemployment levels in counties comes from [48] and [52]. The three measures of education are 1) percent of adults with only a high school diploma, 2) percent of adults completing some college or associate degree, and 3) percent of adults with a bachelor’s degree or higher. Excluded is the percentage of adults with less than a high school diploma, which serves as the baseline. The measure of employment is the unemployment rate.

#### Poverty Data (PD)

Data on poverty levels in counties comes from [50] and [54]. The three measures of poverty are 1) percent of people of all ages living in poverty, 2) percent of people ages zero to seventeen years living in poverty, and 3) percent of people ages five to seventeen years living in poverty.

#### Age Data (AD)

Data on age distributions within a county come from [47] and [51]. This dataset includes the number of people in each of eighteen different age groups. The first seventeen age groups are five years wide (zero to four years, five to nine years, etc.) and the final age group is 85 years or older. We use the data to calculate median age dummy variables. For each county, we calculate the five-year range in which the median person’s age falls. There are ten ranges that have at least one county’s median age. We create a set of nine dummy variables that take the value of one if the county’s median age is within that five-year range and zero otherwise. The first five-year range is used as the baseline.

#### Income Data (ID)

Income data within a county comes from [49] and [53]. The two measures of income are 1) the median household income, and 2) the median household income as a percent of the state total.

### Urban/Rural Data (URD)

The two measures of whether a county is urban or rural are 1) the rural-urban continuum code from 2013 [56], and 2) the urban influence code from 2013 [57]. We use the 2013 versions of these variables as the next update to the data was in 2023, which is well after the pandemic ended. The data was downloaded on March 7, 2025.

### Plot.ly County geojson Data (PCG)

The PCG is a set of JSON files openly provided by Plot.ly. The data allows the mapping of numerical values onto the U.S. map by county or state. This data was used only for visualization purposes. The data is available at https://raw.githubusercontent.com/plotly/datasets/master/geojson-counties-fips.json.

## Analysis and results

This section presents the results of our analysis. Each subsection first describes the method used and then presents the results visually. When feasible, we also include regression tables. Some analyses result in hundreds or thousands of coefficients or are the combination of many individual regressions. In these instances, we refer the reader to the link in the Data Availability section which contains the regression tables and/or Excel exports of the coefficients along with all replication code and data.

Table 1 describes the primary metrics we use in our analysis and highlights the source of the data used to construct the metric.

**Table 1:** Variable definitions

Variable	Definition
$cc_{i,t}$	Cumulative number of COVID-19 cases on day *t*
$VoteMargin_i$	Republican vote margin in county *i*: $Republican~vote~percent - Democrat~vote~percent$
$GR_{i,t}$	COVID-19 case growth rate in county *i* from day $t-1$ to day *t*: $\ln (cc_{i,t}+1)-\ln (cc_{i,t-1}+1)$
$\overline{GR_{i,B}}$	Average COVID-19 case growth rate in county *i* before the national travel ban (March 1, 2020 to March 14, 2020)
$\overline{GR_{i,A}}$	Average COVID-19 case growth rate in county *i* after the national travel ban (March 22, 2020 to April 4, 2020)
$IM_{i,t-l}$	*l*-day lagged individual mobility: $IM = 100 - social~distancing~index$
$\overline{IM_{i,B}}$	Average individual mobility for county *i* before the national travel ban (March 1, 2020 to March 14, 2020)
$\overline{IM_{i,A}}$	Average individual mobility for county *i* after the national travel ban (March 22, 2020 to April 4, 2020)
$TestingCapacity_{i,t-l}$	*l*-day lagged testing capacity
$\overline{TestingCapacity_{i,B-l}}$	Average l-day lagged testing capacity for county *i* before the national travel ban (March 1, 2020 to March 14, 2020)
$\overline{TestingCapacity_{i,A-l}}$	Average l-day lagged testing capacity for county *i* after the national travel ban (March 22, 2020 to April 4, 2020)
$\Delta TestingCapacity_{i,l}$	Change in average l-day lagged testing capacity for county *i* after the national travel ban the national travel ban (March 22, 2020 to April 4, 2020) relative to before the national travel ban (March 1, 2020 to March 14, 2020): $\overline{TestingCapacity_{i,A-l}} - \overline{TestingCapacity_{i,B-l}}$
$\alpha _i$	County-level fixed effects to control for county-level heterogeneity where possible
$\delta _t$	Date-level fixed effects to control for date-level heterogeneity where possible and allow for estimation of time-varying correlations between $T_i$ and $IM_{i,t-l}$

### Reduction in individual mobility correlates with controlling the spread of the pandemic

We used weighted least squares regression to examine whether there is an association between the COVID-19 case growth rate in each county across the US and individual mobility. In particular, we estimated the following specification:

1 $$\begin{aligned} GR_{i,t}=\alpha _i + \delta _t + \beta \times IM_{i,t-14}+\gamma TestingCapacity_{i,t-14}+\epsilon _{i,t} \end{aligned}$$

where *i* indexes counties and *t* indexes days. $GR_{i,t}=\ln (cc_{i,t}+1)-\ln (cc_{i,t-1}+1)$ measures the COVID-19 case growth rate as a percentage change in the cumulative number of cases, where $cc_{i,t}$ is the cumulative number of COVID-19 cases on a given day, and $cc_{i,t-1}$ is the cumulative number of COVID-19 cases on the previous day. One was added to the cumulative number of COVID-19 cases to account for issues when $cc_{i,t}$ is zero, which would make the log undefined. This use of the change in the natural log results in a percentage change which allows for direct comparison across counties of different populations. $IM_{i,t-14}$ is an individual mobility metric from fourteen days prior to the focal day. To compute individual mobility, we reversed the social distancing index in the UMDC19 data: $IM = 100 - social~distancing~index$. An individual mobility score of zero essentially means that all the residents in a county are staying home and no non-residents are entering the county, while an individual mobility score of 100 means that there is no social distancing in the county. $TestingCapacity_{i,t-14}$ controls for a county’s ability to perform COVID-19 tests fourteen days prior to the focal day. $\alpha _i$ is a set of county-level fixed effects that control for any time-invariant characteristics of a county, and $\delta _t$ is a set of date-level fixed effects that control for any characteristics that impact all counties on a given day. The coefficient $\beta$ is of particular interest as it quantifies the relationship between COVID-19 growth rates on a given day and the 14-day lagged individual mobility across all counties and days, controlling for county and time effects. The term $e_{i,t}$ represents residual error. The errors were clustered at the state level to allow for arbitrary correlation across counties and days within a state and heteroscedastic error variances across states [25]. Weights are determined by the population of the county such that counties with larger populations are weighted more heavily in the regression.

**Table 2:** Individual mobility correlates with COVID-19 case growth rate

	(1)	(2)	(3)
Intercept	$$-0.0609^{***}$$	$$-0.0369^{***}$$	$$-0.0636^{***}$$
	(0.0073)	(0.0099)	(0.0085)
$$IM_{t-14}$$	0.0016$$^{***}$$		
	(0.0001)		
$$TestingCapacity_{t-14}$$	0.0003$$^{***}$$		
	(0.0001)		
$$IM_{t-7}$$		0.0012$$^{***}$$	
		(0.0001)	
$TestingCapacity_{t-7}$		0.0001	
		(0.0001)	
$$IM_{t-21}$$			0.0017$$^{***}$$
			(0.0001)
$TestingCapacity_{t-21}$			0.0002
			(0.0001)
Observations	491064	512820	469308
N. of groups	3108	3108	3108
Adjusted $R^2$	0.266	0.266	0.262

The results in Column 1 of Table 2 show an aggregate association between the COVID-19 case growth rate and 14-day lagged individual mobility of 0.0016 (p-value < 0.001). In other words, for the United States as a whole, a one-point increase in individual mobility (out of 100) is associated with an increased COVID-19 cases growth rate of 0.16%. This result is consistent with findings of a relation between individual mobility and COVID-19 cases growth rate reported by [18]. We find similar results using lags of different lags for individual mobility and testing capacity (Columns 2 and 3 of Table 2).

**Fig. 1:**
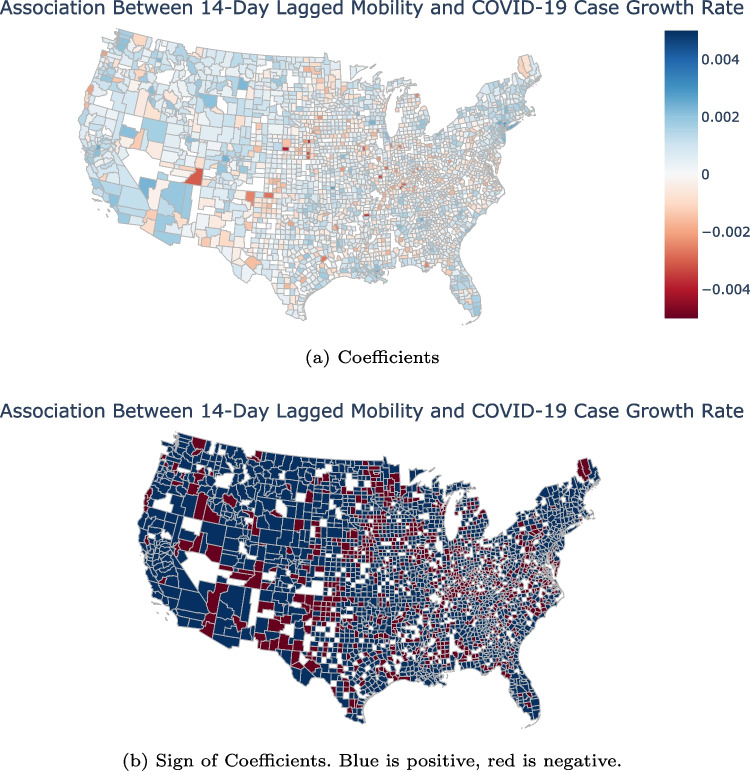
A positive association between increased 14-day lagged individual mobility and COVID-19 cases growth rate. Panel (a) displays the coefficients $\beta _i$ from Eq. 2. Coefficients $\beta _i$ with p>0.05 were set to zero (white) using error clustering at the state level to allow for arbitrary correlations across counties (and days) within each state and heteroscedastic error variances across states [25]. Panel (b) shows in blue the counties with a positive association between 14-day lagged individual mobility and COVID-19 cases growth rate, and in red the counties with a negative association. Counties lacking statistical significance are shown in white

The results based on Eq. 1 demonstrate that, on average, there is a positive association between COVID-19 case growth rates and individual mobility. Moreover, we explored whether there are different associations for each county with the following specification:

2 $$\begin{aligned} GR_{i,t}=\alpha _i + \delta _t + \beta _i \times IM_{i,t-14}+\gamma TestingCapacity_{i,t-14}+\epsilon _{i,t} \end{aligned}$$

where all variables are as described in Eq. 1. The difference between the two equations is the flexibility of Eq. 1 to estimate a different correlation for each county while Eq. 1 assumes the same relationship for all counties. The $\beta _i$ coefficients are of particular interest as they show the relationship between COVID-19 growth rates and individual mobility for each county individually. The results from Eq. 2 show substantial variability in the association between individual mobility and COVID-19 case growth rates across the U.S. counties (Fig. 1). For 66.8% of counties, the association was positive, indicating that increased individual mobility was associated with an increase in the COVID-19 growth rate. We restricted this to only coefficients which had a p-value below 0.05 and found that 69.2% of those counties had a positive association. The associations varied greatly, with the range from −0.0039 to 0.0036. Put another way, while in New York County, New York a one-point increase in individual mobility (out of 100) is associated with an increased COVID-19 cases growth rate of 0.36%, in Dawson County, Nebraska that same one-point increase is associated with a decreased COVID-19 cases growth rate of 0.39%. Figures 5 and 6 in the Appendix show similar results when 4-day and 21-day lags are used for individual mobility and testing capacity.

### Individual mobility is associated with political leaning

After observing the profound variation in the association between individual mobility and COVID-19 cases growth rate, we were interested in understanding possible underlying explanations for such variation. We set out to understand whether variations in political leaning across counties could account for part of the variance in the data. This was a plausible and particularly interesting question given the polarized state of American politics and correspondingly divided media reporting on the COVID-19 pandemic. Such a divide may have had an influence on individuals’ responses to the national travel ban. In particular, we were interested in measuring whether individual mobility was associated with political leaning. Did individuals with different political leaning in the 2016 elections respond differently to the national travel ban?

**Fig. 2:**
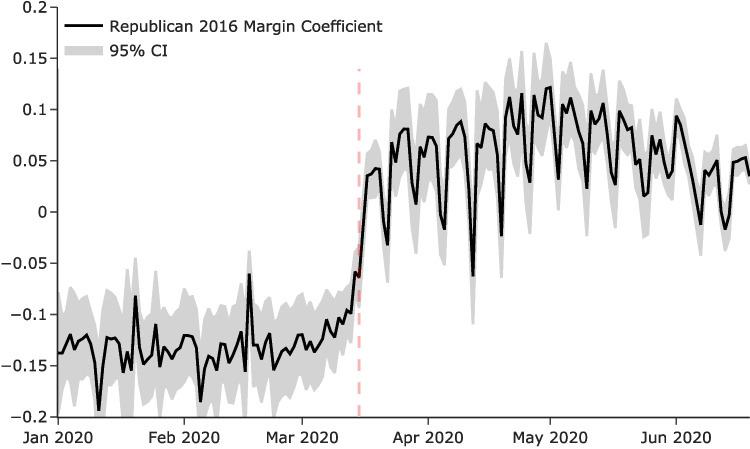
Change in individual mobility correlation with Republican margin in the 2016 elections. The plot shows coefficients $\beta _t$ of Eq. 3 with 95% confidence intervals based on errors clustered at the state level

To do so, we performed a weighted linear regression to estimate the association between individual mobility and the 2016 Republican margin across counties for each day from January 1, 2020 to June 20, 2020:

3 $$\begin{aligned} IM_{i,t}=\alpha _i +\delta _t + \beta _t\delta _t VoteMargin_i + \epsilon _{i,t} \end{aligned}$$

where *i* indexes a county and *t* references a day, $VoteMargin_i$ is the Republican margin in the 2016 election, and all other variables are the same as in Eq. 1. The U.S. county fixed effects ($\alpha _i$) capture time-invariant differences between counties including the direct effect of Republican margin from the 2016 election. The date fixed effects ($\delta _t$) control for any temporal shocks that impact all counties at the same time, such as on weekends or national holidays. The coefficients on the date fixed effects interacted with the Republican 2016 margin ($\beta _t$) capture the correlation between the 2016 Republican margin and daily individual mobility. Errors are clustered at the state level to allow for arbitrary state-level heteroskedasticity and correlated errors within states across all counties and all dates. As before, county populations determine the weights of the observations.

Figure 2 shows the $\beta _t$ coefficients together with their 95% confidence intervals. The results demonstrate a negative association was measured between individuals’ mobility and the 2016 Republican margin before the national travel ban of March 14, 2020. Critically, after the national travel ban, the association quickly grew positive and remained strong through the end of June 2020. The results show that individuals with different political orientations behaved differently in response to the national travel ban. Figure 7 in the Appendix shows that the results are consistent when using the 2020 Republican margin. As these regressions result in a large number of coefficients, the tables are not included in the paper. Instead, we include them in OSF; see the data availability statement for a link and additional information.

**Fig. 3:**
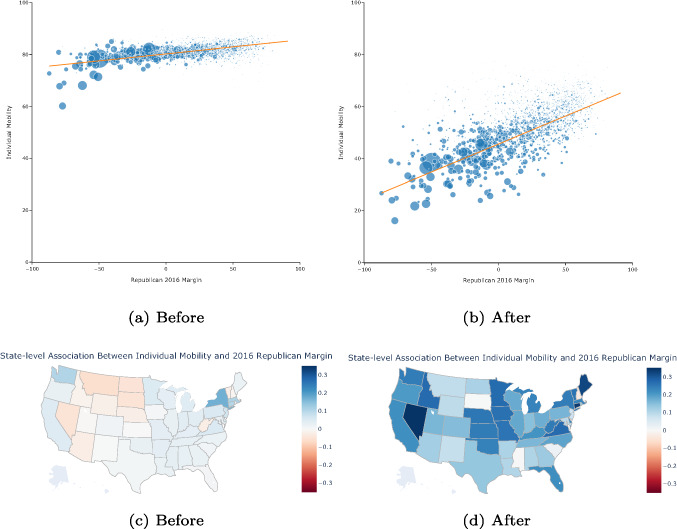
County and state-level association between individual mobility and 2016 Republican margin before and after the national travel ban. County-level individual mobility and Republican margin before (panel a) and after (panel b) the travel ban. We show the trend line showing that the relation between mobility and political leaning goes from zero (baseline; left) to significant after March 14 (right). On this date the national travel ban was issued. The size of each dot represents the population of the county. State-level distribution of the association between individual mobility and Republican margin before (panel c) and after (panel d). Within a state slope of the orange lines. A darker blue means a higher association. It is darker blue after the travel ban

We further described how the relationship between individual mobility data and 2016 Republican margin varies at the county and state levels, both before and after the national travel ban. To do so we estimated the following weighted least squares regression:

4 $$\begin{aligned} \overline{IM_{i,\bullet }} = \alpha + \beta VoteMargin_i + \epsilon _i \end{aligned}$$

where $IM_{i, \bullet }$ is the average individual mobility before the national travel ban (defined as March 1, 2020 to March 14, 2020; denoted by *B* in place of $\cdot$) and after the national travel ban (defined as March 22, 2020, to April 4, 2020; denoted by *A* in place of $\cdot$). Coefficients are estimated separately for the before and after periods. $VoteMargin_i$ is the Republican 2016 margin, and the associated $\beta$ coefficient demonstrates how the average individual mobility correlated with Republican 2016 margin before and after the national travel ban. The county population is used for the weights in the regression.

**Table 3:** Individual mobility is associated with political leaning

	(1)	(2)	(3)	(4)
Republican 2016 Margin	0.054$$^{***}$$	0.215$$^{***}$$		
	(0.001)	(0.004)		
Republican 2020 Margin			0.053$$^{***}$$	0.225$$^{***}$$
			(0.001)	(0.004)
Intercept	80.142$$^{***}$$	45.569$$^{***}$$	80.249$$^{***}$$	46.056$$^{***}$$
	(0.047)	(0.129)	(0.048)	(0.127)
Observations	3108	3108	3108	3108
Adjusted $R^2$	0.334	0.516	0.313	0.538

Figure 3a and b visually demonstrate this regression where all county-days are used to estimate Eq. 4. The scatter points are sized by the population. We note a general shift downwards when moving from Fig. 3a to b indicating that individual mobility generally decreased throughout the country. However, Republican counties adjusted their mobility down less than Democrat counties. Indeed, the average (unweighted) *IM* for counties where the Republican 2016 margin is positive went from 80.95 before to 58.76 after while in the counties where the Republican 2016 margin was negative *IM* went from 80.05 to 49.51. This larger reduction in Democrat-leaning counties aligns with the developing correlation observed in Fig. 7. Similar numbers are found when performing this analysis using the 2020 election numbers instead.

The lines in Fig. 3a and b are weighted least squares regression lines. The slope goes from 0.054 in the before period to 0.215 in the after period. See Table 3 for the full regression results. Figure 8 in the Appendix shows that the results are consistent when using the 2020 Republican margin.

We further looked at how these associations varied across the United States. To do so, we repeated the above process individually forty-eight times, one time per state (excluding D.C. because it only has one county as well as Alaska and Hawaii due to a lack of data). The slope of the line in the weighted least squares regression was then used to color the state in the filled maps in Fig. 3c and d. Consistent with the previous plot, no clear relationship between individual mobility was found before the national travel ban. In contrast, the state-wide association was strong after the travel ban. Critically, this association varied largely across states with a minimum of −0.029 in New Hampshire and a maximum of 0.345 in Nevada. While each of these regressions is small, there were a total of 192 regressions completed (48 states X before, after X 2016, 2020) and as such the tables are not included in the paper. Instead we include them in OSF; see the data availability statement for a link and additional information.

### Politics is associated with changes in the COVID-19 case growth rate

**Table 4:** Politics is associated with changes in COVID-19 case growth rate

	(1)	(2)	(3)	(4)
Republican 2016 Margin	0.0012$$^{***}$$	0.0007$$^{**}$$		
	(0.0003)	(0.0003)		
Republican 2020 Margin			0.0012$$^{***}$$	0.0006$$^{**}$$
			(0.0003)	(0.0003)
$$\Delta TestingCapacity_{i,14}$$		0.0006$$^{**}$$		0.0006$$^{**}$$
		(0.0003)		(0.0003)
Intercept	0.0918$$^{***}$$	0.0783	0.0942$$^{***}$$	0.1171
	(0.0080)	(0.1369)	(0.0078)	(0.1331)
Controls	No	Yes	No	Yes
Observations	3108	3107	3108	3107
Adjusted $R^2$	0.160	0.425	0.149	0.428

Having shown a relationship between COVID-19 case growth rates and individual mobility and a relationship between individual mobility and Republican’s 2016 margin, we then explored the association between COVID-19 case growth rates and Republican’s 2016 margin. In particular, we estimated the following model using weighted least squares:

5 $$\begin{aligned} \overline{GR_{i,A}} - \overline{GR_{i,B}}= \alpha + \beta VoteMargin_i + \epsilon _i \end{aligned}$$

where *i* indexes a county, *B* (*A*) indexes the period before (after) the travel ban, $\overline{GR}$ is the COVID-19 case growth rate, and the bar indicates an average over the days in the before or after period. Consistent with previous analysis, we define before the national travel ban as March 1, 2020 to March 14, 2020 and after the national travel ban as March 22, 2020, to April 4, 2020. $VoteMargin_i$ is the Republican margin in the 2016 election. As before, we use the county population for the regression weight.

**Fig. 4:**
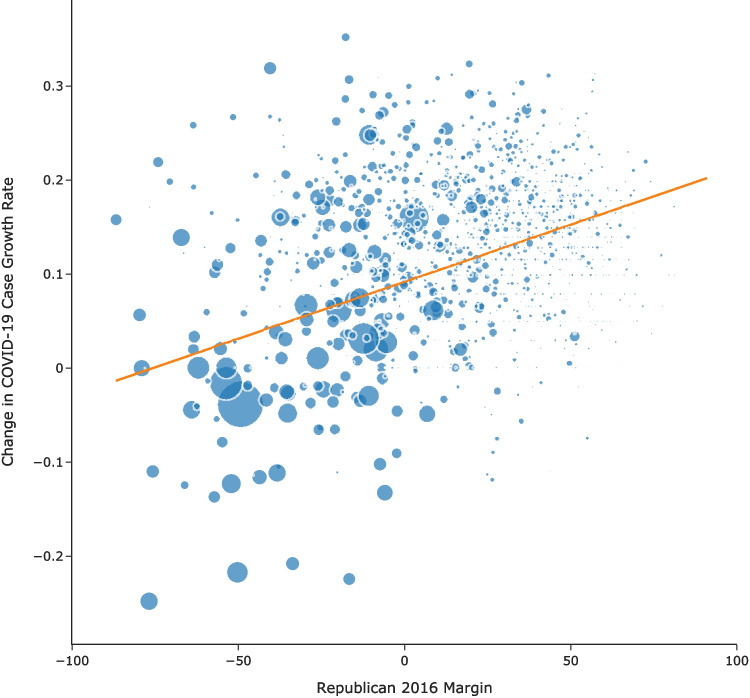
A positive association between the change in COVID-19 case growth rate and 2016 Republican margin. The change in COVID-19 case growth rates after the travel ban relative to that county’s level before the travel ban is positively associated with Republican’s 2016 margin. The size of each dot represents the population of the county

**Fig. 9:**
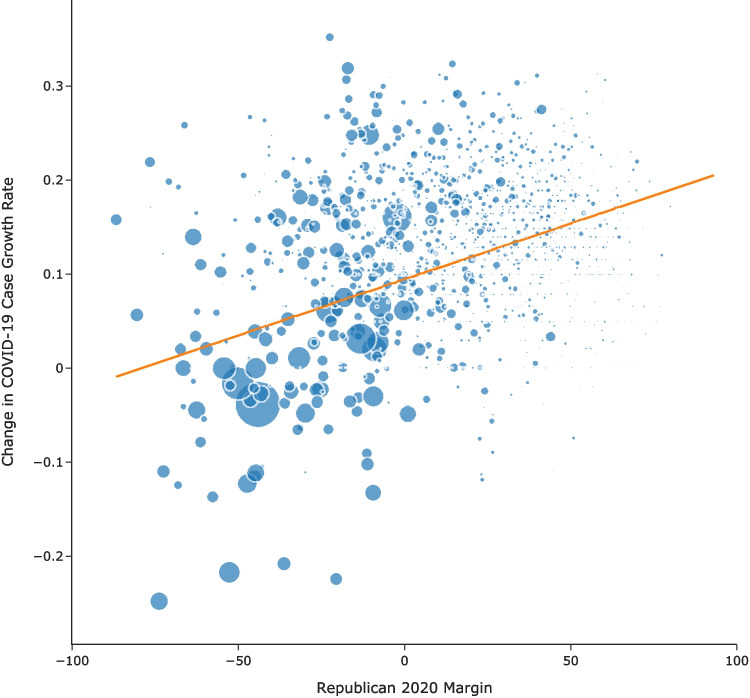
A positive association between the change in COVID-19 case growth rate and 2020 Republican margin. The change in COVID-19 case growth rates after the travel ban relative to that county’s level before the travel ban is positively associated with Republican’s 2020 margin. The size of each dot represents the population of the county

Results in Column 1 of Table 4 show a positive association between Republican’s 2016 margin and the difference in COVID-19 case growth rates before and after the national travel ban (Fig. 4). An example may help with interpreting the Republican 2016 Margin in Column 1 of Table 4 (0.0012). Recall that the COVID-19 growth rate is the percent change in COVID-19 cases so an increase of 0.0012 equates to a 0.12% difference in the COVID-19 growth rate and $VoteMargin_i$ measures the net Republican margin in the election measure as percent Republican minus percent Democrat votes in a county. For a county with a large Republican vote margin of 20%, we would expect to have a COVID-19 growth rate that is $0.12\% \times 20 = 2.4\%$ higher than a similar county with a 50-50 split between Republican and Democrat votes. Column 3 of Table 4 demonstrates that the results are robust to using Republican’s 2020 election margin. Figure 9 in the Appendix shows consistent results using Republican’s 2020 margin.

#### County-level control variables do not change the results

To further test the robustness of this finding, we added control variables to the weighted least squares regression:

6 $$\begin{array}{l}\overline{GR_{i,A}}-\overline{GR_{i,B}}=\\\alpha+\beta VoteMargin_i+\gamma_1\Delta TestingCapacity_{i,14}+\gamma_2X_i+\epsilon_i\end{array}$$

where $\overline{GR_{i,A}}$, $\overline{GR_{i,B}}$, and $VoteMargin_i$ are as previously defined and the regression weights are based on the population in the county. $\Delta TestingCapacity_{i,14}$ = $\overline{TestingCapacity_{i,A-14}}$ – $\overline{TestingCapacity_{i,B-14}}$ controls for the change in average 14-day lagged testing capacity between the two weeks before the national travel ban to the two weeks before the national travel ban. The additional control variables in $X_i$ used were: population (UMDC19), population density (UMDC19), percent of adults with only a high school diploma (EdEmD), percent of adults completing some college or associate degree (EdEmD), percent of adults with a bachelor’s degree or higher (EdEmD), the rural-urban continuum code (URD), the urban influence code (URD), percent of people of all ages living in poverty (PD), percent of people aged zero to seventeen years living in poverty (PD), percent of people aged five to seventeen years living in poverty (PD), nine dummy variables that take the value of one if the county’s median age is within that five-year range and zero otherwise (AD), the unemployment rate (EdEmD), the median household income (ID), and the median household income as a percent of the state total (ID). Results in Column 2 of Table 4 show that the association between the change in COVID-19 case growth rate and Republican’s 2016 margin was significant even after controlling for all these variables. Column 4 of Table 4 shows that the results are robust to using Republican’s 2020 election margin.

## Discussion

The unprecedented gravity of the COVID-19 pandemic mobilized governments to design a series of ad-hoc policies that can limit citizens’ mobility and freedom at multiple levels. Many of these policies involve limiting access to basic infrastructure, public offices, schools, and restrict access to travel [31]. In the U.S., over 90% of the adult population had to change their lifestyle due to the pandemic [44]. Even with vaccines, behavioral remedies, such as limiting individual mobility, can be helpful strategies to reduce the impact of COVID-19.

To truly understand how to limit the spread of the disease, it is vital to go beyond government mandates and policies [16] and capture the multifaceted factors that may influence human behavior and the response to policies. One factor of interest is the relationship between political leanings and social distancing behavior. Much like the severity and policies to deal with the pandemic varied across the U.S., individual compliance with social distancing policies varied across states and often along political lines. This geographical variation begs the question: were conservatives and liberals in the U.S. complying with the social distancing efforts to the same degree? These efforts include reductions in individual mobility and social activities perceived by many as a reduction in individual freedom. This line of reasoning was perhaps best expressed by the U.S. Surgeon General, the leading spokesperson on matters of public health of the U.S. federal government, “Some feel face coverings infringe on their freedom of choice- but if more wear them, we will have more freedom to go out” [34]. Indeed, a national poll reported that the percentage of Democrats who wore a mask all the time when leaving home was approximately 65% between May 8, 2020, and June 22, 2020, whereas the percentage of Republicans who wore masks was just 35% [8].

The divide in the political environment in the United States in early 2020 may suggest that political leaning may play a role in shaping the response to social distancing prescriptions and indirectly affect the spread of COVID-19. First, the U.S. federal government sent mixed signals throughout the pandemic [5]. While official administration guidelines asked Americans to reduce mobility, comply with local laws, and wear masks in public spaces to slow the spread of the pandemic, federal, state, and local officials sent mixed signals [29, 30]. Moreover, media coverage of the severity of the COVID-19 pandemic and addressing the need for social distancing also varied widely along partisan lines. Indeed, U.S. citizens were starkly divided on the perception of the severity of the pandemic since the beginning of the outbreak. Public discourse from top political leaders, as well as the diversity of opinions expressed by the media, might have played a role in this divide. For example, according to the Pew Research Center, in February 2020, 66% of Democratic-leaning citizens believed the news media coverage of COVID-19 was “largely accurate,” while only 31% of Republican-leaning citizens did [24]. This, along with comments in social media from prominent figures may explain why in early March, approximately 40% of Republicans were “not at all concerned” about an outbreak in their communities, whereas less than 5% of Democrats were in that category [9]. To make matters worse, U.S. citizens were also prey to disinformation campaigns regarding COVID-19 from foreign actors, according to the U.S. Department of Defense [23]. All these elements taken together make the inclusion of political beliefs in disease growth models relevant, if not necessary.

While we find that limiting individual mobility was associated with reductions in the COVID-19 growth rate, we confirm that people are heterogeneous in the extent to which they adhered to the government policies and social norms, which is associated with varying levels of case growth rates. Furthermore, we demonstrate with county-level data that this association between growth rates and individual mobility is itself correlated with political leaning such that stronger Republican political leaning is associated with higher COVID-19 growth rates.

Understanding how political beliefs may be associated with particular behaviors is crucial for developing and evaluating policies to reduce mobility but also may help to reopen the economy more quickly following the next pandemic. Our findings may be particularly relevant in certain rural regions that tended Republican in 2016 and where the first wave of COVID-19 may have been delayed [14]. Carefully tracking case growth rates in these areas and pro-actively developing messaging that can influence and motivate human behavior in rural regions may be crucial to reducing the spread of the next pandemic.

## Figures and Tables

**Fig. 5:**
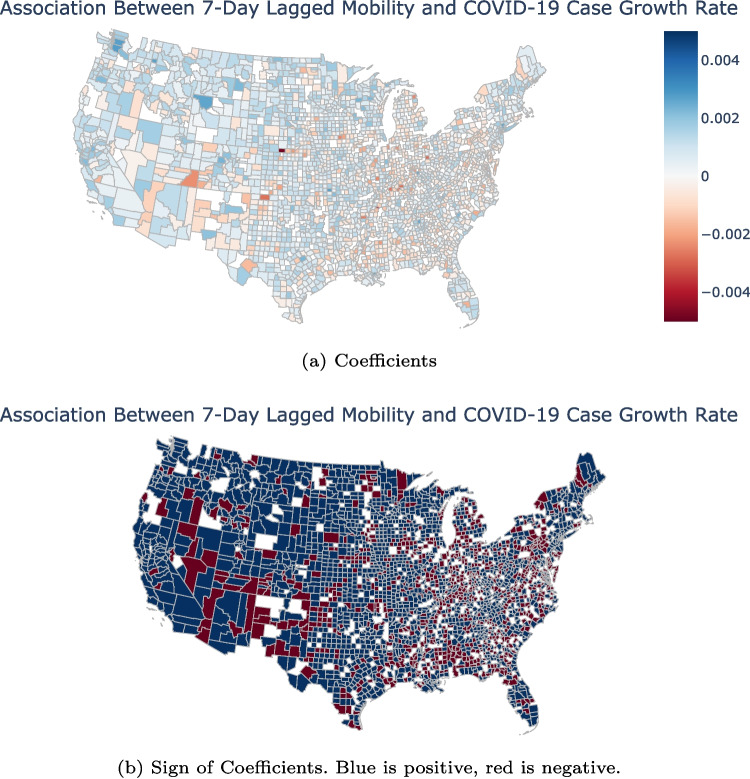
A positive association between increased 7-day lagged individual mobility and COVID-19 cases growth rate. Panel (a) displays the coefficients $\beta _i$ from Eq. 2. Coefficients $\beta _i$ with p>0.05 were set to zero (white) using error clustering at the state level to allow for arbitrary correlations across counties (and days) within each state and heteroscedastic error variances across states [25]. Panel (b) shows in blue the counties with a positive association between 7-day lagged individual mobility and COVID-19 cases growth rate, and in red the counties with a negative association. Counties lacking statistical significance are shown in white

**Fig. 6:**
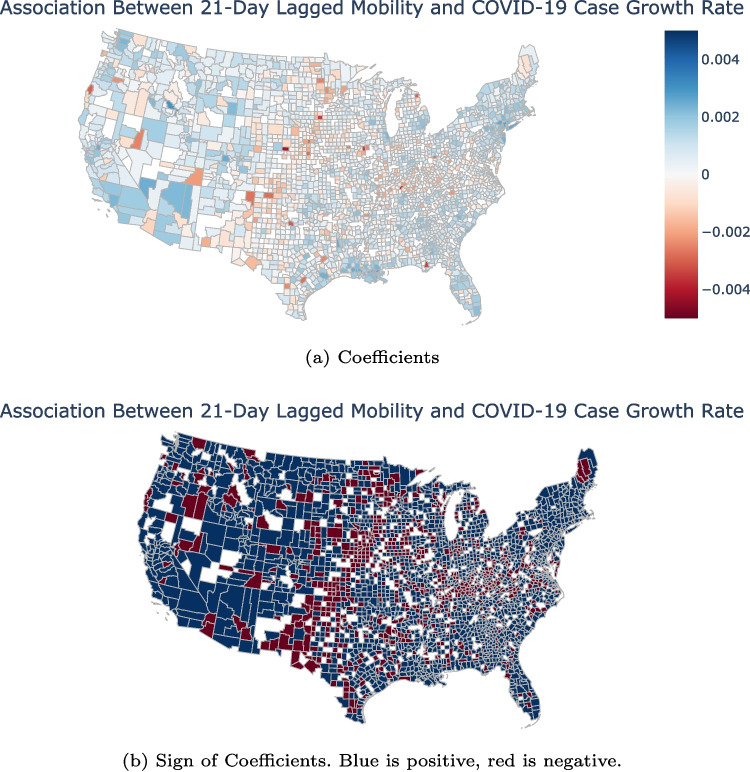
A positive association between increased 21-day lagged individual mobility and COVID-19 cases growth rate. Panel (a) displays the coefficients $\beta _i$ from Eq. 2. Coefficients $\beta _i$ with p>0.05 were set to zero (white) using error clustering at the state level to allow for arbitrary correlations across counties (and days) within each state and heteroscedastic error variances across states [25]. Panel (b) shows in blue the counties with a positive association between 21-day lagged individual mobility and COVID-19 cases growth rate, and in red the counties with a negative association. Counties lacking statistical significance are shown in white

**Fig. 7:**
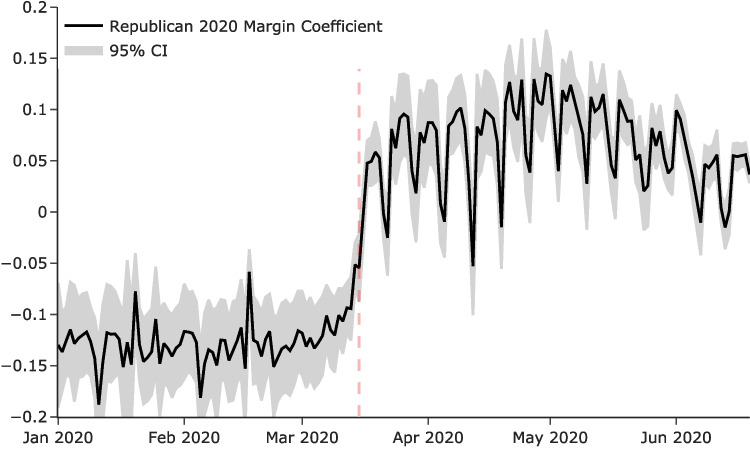
Change in individual mobility correlation with Republican margin in the 2020 elections. The plot shows coefficients $\beta _t$ of Eq. 3 with 95% confidence intervals based on errors clustered at the state level

**Fig. 8:**
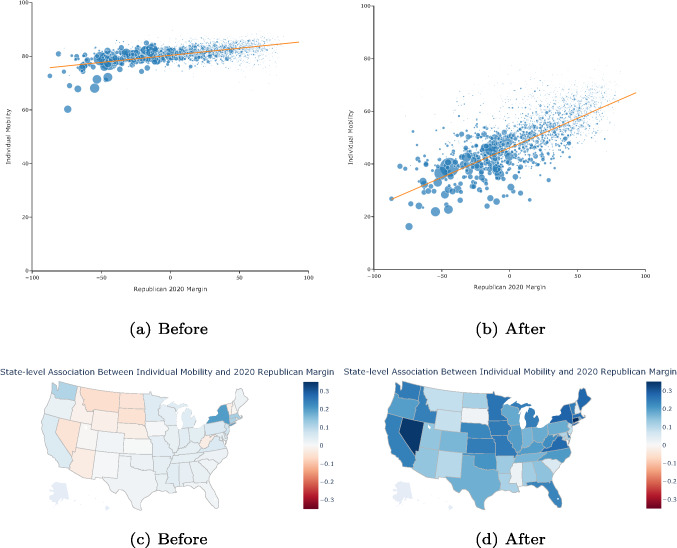
County and state-level association between individual mobility and 2020 Republican margin before and after the national travel ban. County-level individual mobility and Republican margin before (panel a) and after (panel b) the travel ban. We show the trend line showing that the relation between mobility and political leaning goes from zero (baseline; left) to significant after March 14 (right). On this date the national travel ban was issued. The size of each dot represents the population of the county. State-level distribution of the association between individual mobility and Republican margin before (panel c) and after (panel d). Within a state slope of the orange lines. A darker blue means a higher association. It is darker blue after the travel ban

## Data Availability

All the data used and generated for this paper is publicly available, the sources are reported above. All the data extracted from the original sources and the code used to organize and analyze the data is publicly available as a Jupyter Notebook at https://osf.io/dpsja/.
